# The General Practitioner’s Part in the Oral Health Propaganda

**Published:** 1915-03

**Authors:** Harold Clark

**Affiliations:** Toronto, Can.


### THE GENERAL PRACTITIONER'S PART IN THE ORAL HEALTH PROPAGANDA.

BY HAROLD CLARK, D.D.S., TORONTO, CAN.

*Note*—This is an extract from a paper read by Dr. Clark last summer before the New Jersey Dental Society and which is printed in full in *Oral Health* for February, under the title, *The New Gospel of Health According to the Dentist.*

After referring to some of the work done by noted dental science workers, and the facts they have developed in regard to the causes and periods of immunity, he gives his view of what the thoughtful general practitioner should attempt to help on the cause.

The bulk of the problem, of course, has yet to be worked out by the investigations of the men of science, but recent research has given us many new conclusions, some possibly correct, others probably correct, and still others as well established as the limitations of laboratory methods will allow. Surely an important part of the work lies at the door of the practicing dentist. It is he who can try out and check up the conclusions of the laboratory man. If each one of us could experiment on even a few actual cases and report results to some central bureau, a mass of practical information would accumulate, from which many positive and valuable deductions might be made, and many a theory would either become a law or be set aside as untenable. It seems to me that the opportunity for the dentist to do a great service to humanity is very large. In his endeavor to lay his finger on the real cause of caries susceptibility, it seems more than probable that he is also discovering the real cause of many of the ailments and diseases that beset the lives of civilized humanity, and make for physical degeneracy.

If this metabolic carbohydrate that is found in the mouth, in the blood stream, in the very tubules of the dentine of carious teeth, is responsible for the rapid proliferation of many varieties of germs found in the mouth, what reason have we for thinking that its influence is confined to the mouth? Is it not probable that while this fertile pabulum is being poured into the mouth it is also being poured into the whole alimentary tract? May it not be responsible for the high bacterial content of the intestines, which Metchnikoff would combat with a buttermilk diet? May it not account for the high degree of infection so frequent in the appendix, and thereby explain the prevalence of appendicitis? And may it not also explain the susceptibility of one individual to typhoid invasion and the immunity of another who is similarly exposed?

Personally I have done but a fraction of what I might and ought to do along these lines. From the beginning of my experience as a dentist I have been much interested in the enigma of immunity and susceptibility. Although I have been possessed of neither the time nor the training which would enable me to do any scientific research on the problem, yet like any practising dentist, I have had much opportunity for making observations and drawing some conclusions. I have for years enquired into the diet of patients exhibiting the extremes of immunity and susceptibility, and I long ago observed how generally susceptibility went with the excessive ingestion of sugar and sweetened foods. Miller's theory calls for the fermentation of the *debris* of carbohydrate food about the teeth. Now, the solubility of sugar, its constant dilution in the mouth with saliva, and the rapidity with which it must be swallowed convinced me that its baneful action on the teeth must be some reaction after deglutition, but I could get no further until Pickerill, Kirk and others bridged the gap for me. I learn that over-ingestion of carbohydrates resulted in a soluble carbohydrate returning to the mouth by way of the circulation. I learned that this substance was a most fertile culture medium for micro-organic life. On reading some of Pickerill's tables I found that my convictions regarding the culpability of sugar in the causation of caries were put to considerable strain, for he shows that cane sugar is far below chocolate, cake, pastry, and even toast, in the production of lactic acid. I strongly suspect, however, that the mischief done by sugar is as much indirect as direct, but none the less positive. Sugar is so attractive to the average human palate that it, or any food made rich with it, is eaten far beyond the normal promptings of hunger, and thereby the alimentary system is over-supplied with carbohydrate foods of various kinds. Sugar confronts us in so many ways. In candies and chocolate preparations. We dissolve it in our drinks, tea, coffee, cocoa, lemonade, etc. We spread it on our cereals, puddings, pastry and fruit. We cook it into our cakes, pies, puddings, etc., also in jams, jellies and marmalade. We find it in syrups and honey. It is indeed difficult at the ordinary table to avoid sugar and sweetened food, even when one wishes to, and, as I said before, it leads to the over-consumption of carbohydrate food. A homely example will point my argument. A hungry individual will eat heartily of meat and vegetables until he can be prevailed to take no more, and then he will eat a helping of some sweetened pudding, and perhaps a second helping. If, instead of the sweetened pudding, he had been offered plain boiled rice he would have eaten none of it, proving that the joke about the little boy's definition of a dessert was no joke at all. He said, you will remember, that a dessert was what you eat after you have had enough.

My enquiries as to the diet of immunes and highly susceptibles has made very strong my conviction that caries of the teeth is largely a matter of diet. Frequently I have, for a time, thought I had found cases that contradicted, or were exceptions to, my usual experience, but further enquiry revealed that some point had been overlooked. An example or two will illustrate. I have a patient who is immaculate in the care of his teeth and rigid in his diet according to his lights. For years he exhibited less than usual susceptibility. Recently he presented himself and I found a surprising number of cavities. Upon enquiry there seemed no reason from his diet for the increased susceptibility, but further questions revealed the fact that for some time he had been trying to increase his weight, and, to that end, he was drinking each night a quart of milk, a large part of which he took in the form of hot cocoa. In view of the fact, set forth in one of Pickerill's tables, that chocolate is more than twenty times as fermentable as cane sugar, it was easy to suspect that his cocoa was responsible for his increased susceptibility.

Another case was a man of about 48 years. After unusual immunity for years, he suddenly exhibited several carious teeth. I probed for sugar in his diet. He confidently told me I should have "to guess again," as he wasn't fond of sugar and left the candies to the ladies. I made a detailed enquiry, running over a list of possible sources, naming jams, jellies, marmalade, honey, syrup, etc. At the mention of honey, his wife, who was standing by, exclaimed, "Why, George just lives on honey!" It transpired that some two years previously he had discovered something special in the honey line and was assured that it was most wholesome as a food.

In some cases I have had the patient for one week keep a detailed record of everything eaten or drunk, and some amazing menus have been revealed by this method which never would have come out with the ordinary enquiry. I have to thank Dr. Kirk for suggesting this way of getting at facts.

I shall refrain from a further recital of cases, as they are usually very tedious, but I might tell of several children with marked susceptibility, where I was able to gain the co-operation of both child and parent in the observation of a more correct diet. After the first year these cases have become practically immune, and, with it all, a noticeable improvement in general health.

I have found some difficulty in the matter of instructing patients about a diet. One may tell them to choose the rougher, more fibrous foods that will demand mastication; to cut down their consumption of sugar and sweetened foods; to eat succulent vegetables and fruits, etc. But these are generalities, and many patients don't seem to be able to follow them intelligently. It has taught me the need of having a more specific or detailed diet to recommend.

To this end would it not be possible and practicable to have a few committees in various centres, composed of, say, a dentist posted on the essentials of diet for the well-being of the teeth and mouth; a physician, with special fitness as an alimentary specialist, and the head of a domestic science school, and have these committees work out a week's menu, physiologically correct, and at the same time presenting an agreeable and appetizing variety. Along with this physiological bill of fare might be appended, or paralleled, a list of those foods or combinations that should be avoided. I am well aware that a menu suitable for one would not be best for all. Age, occupation, climate, and other things would call for modifications of a general diet. But the vital principles that are essential for one would probably be the same for all, except perhaps in some pathological cases.

We are all familiar with the work done by the dentist in the public schools of Cleveland and other centres to arouse public interest in the national importance of having the rising generation grow up without the handicap that goes with decaying teeth and foul mouths. It has been abundantly proved that much of the physical degeneracy about us and, with it, mental and moral degeneracy, are traceable to dental caries and septic mouths. If the dentist did no more for the public weal than the carrying out of this work, the results should entitle him to a place among the noble professions; but I am convinced that a far greater opportunity for service is knocking at his door. I am satisfied that our scientific investigators are establishing a positive relationship between a faulty selection and balance of diet and dental caries. I am hopeful that, with the organized co-operation of the practising dentist, it will soon be possible to put practical immunity to caries within the reach of all; and in wiping out the causes of dental caries there is little doubt that with it will disappear many of the maladies that afflict civilized humanity and make for degeneracy.

